# Delivery-Corrected Imaging of Fluorescently-Labeled Glucose Reveals Distinct Metabolic Phenotypes in Murine Breast Cancer

**DOI:** 10.1371/journal.pone.0115529

**Published:** 2014-12-19

**Authors:** Amy E. Frees, Narasimhan Rajaram, Samuel S. McCachren, Andrew N. Fontanella, Mark W. Dewhirst, Nimmi Ramanujam

**Affiliations:** 1 Department of Biomedical Engineering, Duke University, Durham, NC, United States of America; 2 Duke University Medical Center, Durham, NC, United States of America; Mayo Clinic College of Medicine, United States of America

## Abstract

When monitoring response to cancer therapy, it is important to differentiate changes in glucose tracer uptake caused by altered delivery versus a true metabolic shift. Here, we propose an optical imaging method to quantify glucose uptake and correct for *in vivo* delivery effects. Glucose uptake was measured using a fluorescent D-glucose derivative 2-(N-(7-Nitrobenz-2-oxa-1,3-diazol-4-yl)Amino)-2-deoxy-D-glucose (2-NBDG) in mice implanted with dorsal skin flap window chambers. Additionally, vascular oxygenation (SO_2_) was calculated using only endogenous hemoglobin contrast. Results showed that the delivery factor proposed for correction, “R_D_”, reported on red blood cell velocity and injected 2-NBDG dose. Delivery-corrected 2-NBDG uptake (2-NBDG_60_/R_D_) inversely correlated with blood glucose in normal tissue, indicating sensitivity to glucose demand. We further applied our method in metastatic 4T1 and nonmetastatic 4T07 murine mammary adenocarcinomas. The ratio 2-NBDG_60_/R_D_ was increased in 4T1 tumors relative to 4T07 tumors yet average SO_2_ was comparable, suggesting a shift toward a “Warburgian” (aerobic glycolysis) metabolism in the metastatic 4T1 line. In heterogeneous regions of both 4T1 and 4T07, 2-NBDG_60_/R_D_ increased slightly but significantly as vascular oxygenation decreased, indicative of the Pasteur effect in both tumors. These data demonstrate the utility of delivery-corrected 2-NBDG and vascular oxygenation imaging for differentiating metabolic phenotypes *in vivo*.

## Introduction

Due to advances in genetic profiling, a host of targeted therapies has been developed to pinpoint specific mutations in cancer [1], [2]. For example, several drugs have been developed that inhibit PI3K signaling, which is dysregulated in cancers of the breast, colon, and ovary, among others [3]–[6]. Some of these targeted therapies can improve tumor perfusion, and hence, delivery of imaging agents such as FDG, while independently modifying intrinsic glucose demand [7]. On the other hand, highly angiogenic tumors or tumors with aberrant vascular signaling may have limited capacity for nutrient or drug delivery [8]. The limited delivery of FDG, for example, could lead to an incorrect perception that the tumor's demand for glucose is low. It is therefore important to identify whether perceived changes in glucose uptake are caused by vascular or true glycolytic changes.

Clinically, immunohistochemistry (IHC) and 18-FDG Positron Emission Tomography (PET) imaging are widely accepted methods for glucose imaging. IHC can effectively quantify glucose transporters (GLUTs) in tumors [9], but requires labor-intensive *ex vivo* tissue processing and staining. PET imaging is another invaluable clinical tool for measuring glucose uptake in tumors or metastases [10]–[12]. PET offers necessary insight into tumor metabolism, but the method is not without limitations: for example, limited spatial resolution [13] and prohibitive cost.

Additionally, both PET and IHC can inform on oxygenation properties of tissue. IHC can be used to quantify hypoxic fraction via staining with nitroimidazoles (e.g. pimonidazole or EF5) [9], [14], [15], but cannot provide kinetic information due to sample preparation. PET can report on both hypoxia and blood flow to further inform on the tumor microenvironment. PET hypoxia imaging with nitroimidazole compounds exhibit low tumor to background contrast, however [16]. Additionally, either hypoxia or blood flow imaging with PET requires the use of additional tracers, further increasing the complexity and cost of the technique [17], [18].

Like FDG, the fluorescent glucose analog 2-NBDG has been shown to serve as a marker of glucose uptake in a variety of cell and animal models [19]–[24]. Uptake of 2-NBDG can be imaged using a host of optical imaging techniques. These same optical techniques can also be leveraged to measure tumor vascular blood flow and oxygenation without the use of exogenous tracers [25], [26]. Our group has developed an *in vivo* optical imaging strategy that utilizes a combination of 2-NBDG uptake and oxygenation to report on tumor metabolism [27]. We used our imaging strategy in a dorsal skin flap model of murine breast cancers and identified four parameters that describe the tumor vasculature and uptake kinetics of 2-NBDG: vascular oxygenation (SO_2_), rate of delivery of 2-NBDG (R_D_), rate of clearance of 2-NBDG (R_C_), and glucose uptake (2-NBDG_60_). We used these parameters to demonstrate that the delivery kinetics of 2-NBDG *in vivo* have profound effects on uptake and, in turn, perceived glycolytic demand. Several groups have demonstrated a similar phenomenon with FDG-PET, showing that knowledge of blood flow is crucial to interpreting FDG-PET based glucose uptake [18], [28]. For example, Specht and colleagues showed that using the ratio of the metabolic rate of FDG (MRFDG) to blood flow as a surrogate for metabolism was a better indicator of long-term fate than using MRFDG alone [28].

Likewise, we sought here to demonstrate that correcting uptake of 2-NBDG, NBDG_60_, by the rate of delivery, R_D_, showed improved contrast between distinct tumor phenotypes. The first aim of the current study was to demonstrate that the ratio 2-NBDG_60_/R_D_ serves as a delivery-corrected measure of glucose uptake in murine dorsal skin flap window chamber models containing normal tissues and tumors. Importantly, the ratio was able to distinguish specific uptake of 2-NBDG from accumulation of a fluorescent control, 2-NBDLG, which is identical to 2-NBDG in molecular weight and fluorescent spectrum, but is unable to undergo active transport into the cell [29]. The ratio 2-NBDG_60_/R_D_ was then leveraged to compare different tumor phenotypes and to characterize the dependence of glucose uptake on vascular oxygenation within these tumors. Our results showed that 2-NBDG_60_/R_D_ was an effective endpoint for comparing *in vivo* glucose uptake of metastatic 4T1 and nonmetastatic 4T07 murine mammary adenocarcinomas derived from the same spontaneous parental tumor [30]. Further, the addition of vascular information revealed metabolic heterogeneity within the tumors. The results presented here indicate that optical imaging of 2-NBDG/R_D_ and vascular endpoints can reveal interesting and distinct phenotypes in normal tissue and tumors.

## Materials and Methods

### Cell Culture Maintenance and Seahorse Assay

Two murine mammary carcinoma cell lines, 4T1 and 4T07, were used in this study. Though arising from the same tumor, the cell lines have distinct different metastatic potential [31]. 4T1 cells have been shown to metastasize throughout the body to organs such as the lung, liver, bone and brain. 4T07 is able to seed into the lung and liver but it fails to engraft to form metastatic nodules. Both cell lines were cultured in Dulbecco's Modified Eagle Medium (DMEM, Gibco, Carlsbad, California) supplemented with 10% fetal bovine serum and 1% antibiotics and kept free from contaminants. Cells were passaged every 2–3 days and kept incubated at 37.0°C and 5.0% O_2_.

A Seahorse Glycolytic Stress Test [Seahorse Biosciences, Massachusetts, USA] was used to measure the metabolic properties of 4T1 and 4T07 cells. Oxygen consumption rate (OCR) and extracellular acidification rate (ECAR) were measured every 11 minutes. OCR was calculated based on changes in dissolved oxygen in the cell media and ECAR was calculated based on detection of changes in free proton concentration in the cell media. Between minute 22 and minute 33 of the assay, 25 mM glucose was injected to each well. Between minute 55 and minute 66, 1 uM oligomycin was injected to each well. Oligomycin inhibits oxygen consumption used for ATP synthesis through phosphorylating respiration [32]. Results for each well were normalized to the number of cells in each well. Results represent the average of 12 total wells for each cell line: assays were performed on 3 different days and each assay contained 4 replicate wells of each cell line.

### Dorsal Window Chamber Implantation

All animal work was performed according to the recommendations of the Guide for the Care and Use of Laboratory Animals of the National Institutes of Health. The Duke University Institutional Animal Care and Use Committee approved all experiments (Protocol Number: A170-12-06). Female nu/nu athymic mice (NCI, Frederic, Maryland), 8–10 weeks old and weighing between 20–25 g, were used for all *in vivo* studies. Murine dorsal window chambers were implanted according to the sterile procedure detailed by Palmer [33]. Briefly, mice were anesthetized via i.p. administration of ketamine (100 mg/kg) and xylazine (10 mg/kg)) and implanted with a titanium dorsal window chamber (APJ Trading Co, Inc, Ventura, California). For tumor development, a 20 µL suspension (20,000 cells) of 4T1-RFP or 4T07 cells was injected into the dorsal skin fold. No cells were injected into the mice in the normal (non-tumor) group. A glass coverslip (diameter  = 12 mm, No. 2, Erie Scientific, Portsmouth, New Hampshire) was placed in the dorsal chamber to cover the exposed tissue. Animals were housed on-site at Duke University under standard 12-hour light/dark cycles. During housing, all animals were provided *ad libitum* access to food and water.

### Imaging Platform

Our imaging system and procedure have both been described in detail [27], [34]. A Zeiss Axioskop 2 microscope fitted with a 2.5x objective (NA = 0.075) was used for both trans-illumination vascular imaging and epi-illumination fluorescence (2-NBDG) imaging. A liquid crystal tunable filter (LCTF) was used for hyperspectral imaging, and a DVC 1412 CCD camera (DVC Company) recorded all images. Hyperspectral imaging was used for all 2-NBDG, 2-NBDLG, and SO_2_ imaging. Trans-illumination images were acquired from 520 to 620 nm in 10 nm increments and used to calculate SO_2_. A 470 nm bandpass excitation filter (40 nm bandwidth) was used for 2-NBDG/2-NBDLG imaging, with a collection wavelength of 525 nm (10 nm bandwidth). Flow imaging was performed using a Zeiss Axio Observer microscope fitted with a broad-spectrum halogen source. Discrete red blood cells were imaged through a 5x objective (Zeiss FLUAR; 0.25 NA) using a 500–550 nm bandpass filter to maximize endogenous contrast from hemoglobin absorption. For fluorescence imaging, image acquisition times were as follows: 300 ms for the 6 mM 2-NBDG and 2-NBDLG groups, and 200 ms for the 10 mM 2-NBDG group. The SO_2_ absorption images were calibrated for wavelength-dependent variations in throughput using images of a neutral density filter (ND = 2, Thorlabs, USA) acquired at each corresponding wavelength (520 nm–620 nm in 10 nm increments). For calibration of 2-NBDG and 2-NBDLG images, fluorescence intensity of a 90.8 µM rhodamine solution in a petri dish was collected at the integration time used for imaging. The average pixel intensity of the corresponding rhodamine image was then used to linearly scale 2-NBDG and 2-NBDLG images.

### Imaging Procedure

During the 6-hour period prior to imaging, animals were fasted but allowed access to water. Immediately before imaging, blood glucose was measured from the tail vein using a FreeStyle Lite Blood Glucose Meter (Abbott Laboratories, Illinois, USA). Mice were then anesthetized with 2% v/v isoflurane mixed with air, which was reduced to 1–1.5% v/v isoflurane for maintenance. The mouse was kept on a heated stage for the duration of imaging. Prior to 2-NBDG injection trans-illumination images were recorded for vascular characterization. Background fluorescence images corresponding to endogenous fluorescence from cellular FAD and stromal collagen at 525 nm were also recorded prior to injection [35]. A 100 µL injection of 6 mM 2-NBDLG, 6 mM 2-NBDG, or 10 mM 2-NBDG in sterile saline was then administered via tail vein. Fluorescence from the tracer was recorded for 60 minutes: continuously for the first 10 minutes, every 30 seconds for the next 30 minutes and every 3 minutes for the final 20 minutes of imaging.

**Figure 1:**
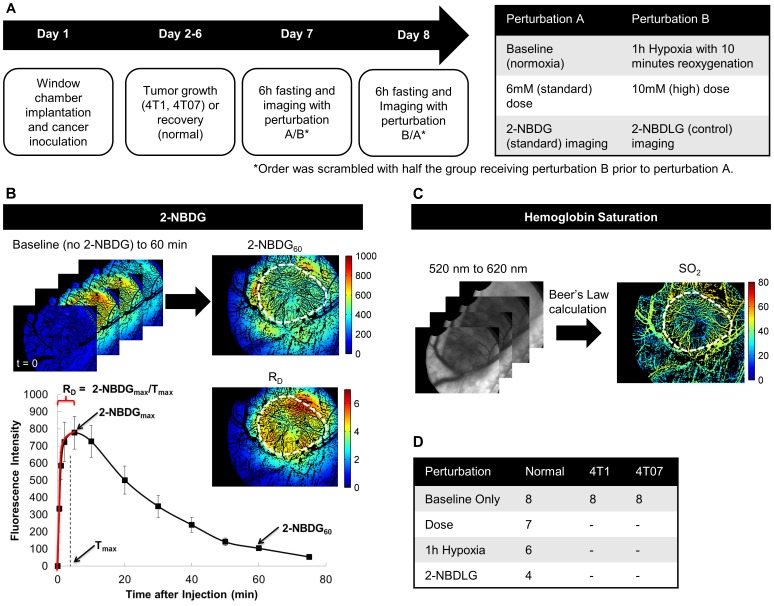
Outline of methods. (A) Timeline of imaging events. Mice that were imaged under two imaging conditions were imaged on subsequent days. The order of imaging was scrambled to minimize order effects. (B) A 6 mM injection of 2-NBDG was given and imaged for at least 60 minutes, and the mean of the tumor region for each image was used to construct a kinetic curve. Images for the endpoints 2-NBDG_60_ (2-NBDG intensity at 60 minutes) and the rate of delivery of 2-NBDG (R_D_ = 2-NBDG_60_/T_max_) are shown. (C) Trans-illumination images were collected in 10 nm increments from 500–600 nm and used to calculate hemoglobin saturation (SO2). (D) The table shows the number of mice used in each perturbation group. Each mouse was used for up to two imaging sessions, with 24 hours between sessions. The groups were randomized to minimize bias from imaging order, and an analysis of variance (ANOVA) was performed to test for order effects. No significant imaging order effect was observed for any experiment.

For the hyperemia study, which was conducted to extend the range of red blood cell velocities, mice were subjected to an hour of breathing hypoxic gas (10% O_2_, balance N_2_) and then allowed to breathe room air for 10 minutes. Imaging began immediately following the 10-minute reoxygenation period using the imaging protocol described above. Mice receiving two perturbations (6 mM and 10 mM, 2-NBDG and 2-NBDLG, or baseline and post-hypoxia imaging) were imaged on two consecutive days to allow for 2-NBDG clearance and ample recovery from anesthesia. At the completion of imaging, mice were euthanized by injectable euthanizing agent (Euthasol, Virbac, USA; 0.05 mL via i.p. injection) in accordance with a protocol approved by The Duke University Institutional Animal Care and Use Committee. An overview of methods is shown in Fig. 1.

### Calculation of Vascular and Metabolic Parameters

Trans-illumination images were collected in 10 nm increments from 500–600 nm and used to create an image cube (x,y,λ). Our procedure was previously described in detail [27]. A modified form of the Beer-Lambert law uses the extinction coefficients of [HbO_2_] and [dHb] to calculate the concentrations of each absorber at each pixel. We then calculate total hemoglobin content, [THb] ([HbO_2_]+[dHb]), and SO_2_ ([HbO_2_]/[THb]) at each pixel. The presence or absence of [THb] was used to segment the images into vascular and tissue space, respectively.

After 2-NBDG injection, fluorescence images were collected) for a period of 75 minutes. A kinetic uptake curve was created from the (x,y,t) data for each (x,y) pixel location. As shown in Fig. 1, the initial rate of delivery (R_D_) and glucose uptake (2-NBDG_60_) were calculated from the time course for each pixel. R_D_ was calculated from the rise to the initial peak of the curve as (I_max_-I_0_)/T_max_, where subscript 0 corresponds to a baseline image captured prior to 2-NBDG injection. 2-NBDG_60_ is defined as glucose uptake. We showed previously that 2-NBDG fluorescence at 60 minutes is confined to the intercellular space [27].

For 4T1 and 4T07 tumors, each endpoint (2-NBDG_60_, R_D_, 2-NBDG_60_/R_D_) was additionally parsed by SO_2_. For each 2-NBDG_60_, R_D_, or 2-NBDG_60_/R_D_ image, every tissue pixel in the tumor area was assigned to an SO_2_ group according to the SO_2_ of the nearest vascular pixel. In a given image, there were as many as five SO_2_ groups: 0–10% SO_2_, 10–20% SO_2_, 20–40% SO_2_, 40–60% SO_2_, and 60–80% SO_2_. The distribution of pixels for each endpoint was then represented as a survival curve (1-cumulative distribution) stratified by SO_2_. Curves were then averaged within a tumor type (4T1 or 4T07). Each curve then represents the mean of distributions of 2-NBDG_60_, R_D_, or 2-NBDG_60_/R_D_ pixels at a given SO_2_ level from up to 8 mice.

The blood flow imaging procedure has previously been described in detail [26]. In short, a video of individual red blood cells flowing through vessels in a non-tumor bearing window chamber was collected, taking advantage of the absorption properties of hemoglobin. A cross-correlation was performed between subsequent frames to track red blood cell movement. For each mouse, we calculated both blood velocity and 2-NBDG delivery (R_D_) in the image region surrounding the vessel with the fastest blood velocity. This allowed us to achieve a wide range of blood velocities over which to correlate blood velocity with R_D_. For a given mouse, the same region was selected in corresponding blood velocity and 2-NBDG images, and kept consistent between days.

### Statistical Analysis

Seahorse assay results were compared with unpaired student's t-tests. Results showing 6 mM and 10 mM doses of 2-NBDG, kinetics at baseline and after hypoxia, or endpoints from 2-NBDG and 2-NBDLG imaging were compared using a student's paired t-test. Each paired test corresponds to the same cohort of mice being imaged on consecutive days under two different imaging parameters. Imaging order was scrambled in all studies- for example, half the mice received 6 mM 2-NBDG on day 1 and 10 mM 2-NBDG on day 2, and half received 10 mM 2-NBDG on day 1 and 6 mM 2-NBDG on day 2. Correlations between parameters were determined by Pearson's linear correlation. For tumor studies containing multiple groups, a one-way analysis of variance (ANOVA) was performed to test for global differences and a Tukey-Kramer post-hoc test was used to compare between groups. Survival curves were compared using repeated measures ANOVA. For all analyses, differences between groups were deemed significant at a 95% confidence level (p≤0.05). The Statistics Toolbox in MATLAB (MathWorks, USA) was used for all statistical tests.

## Results

### Delivery-corrected 2-NBDG-uptake inversely correlates with blood glucose concentration

**Figure 2:**
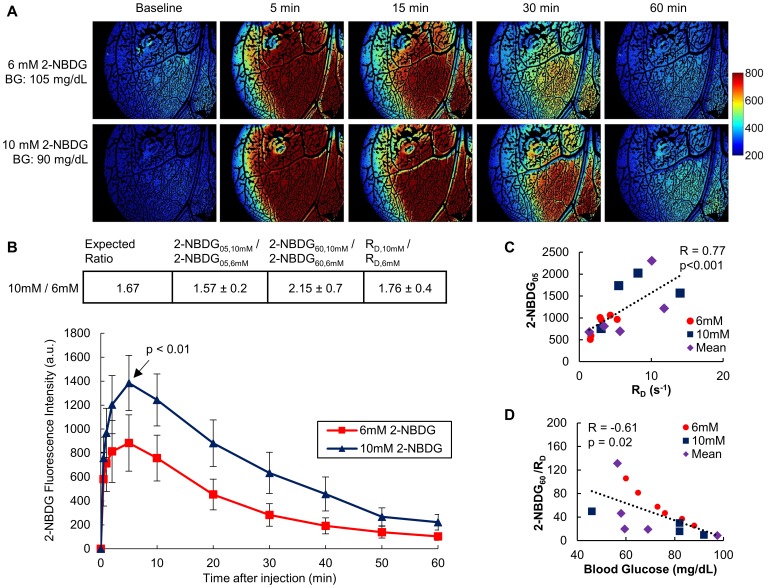
Delivery-corrected 2-NBDG uptake inversely correlates with blood glucose concentration. (A) Representative images show the kinetics of 2-NBDG uptake *in vivo* in non-tumor window chambers. The same mouse was given 6 mM or 10 mM 2-NBDG on subsequent days and imaged for 60 minutes following injection. (B) Averaged 2-NBDG kinetics for a cohort of mice injected with 0.1 mL of either 6 mM or 10 mM 2-NBDG. At 5 minutes post-injection (2-NBDG_05_), the fluorescence ratio of the dose groups (2-NBDG_05,10 mM_/2-NBDG_05,6 mM_) was proportional to molarity (p<0.01). The table shows the expected ratio of 10 mM/6 mM fluorescence, if all differences in fluorescence were due to dose. 2-NBDG_05,10 mM_/2-NBDG_05,6 mM_ corresponds to the ratio of 10 mM and 6 mM fluorescence intensities at t = 5 min. The ratio R_D,10 mM_/R_D,6 mM_ corresponds to the rate of 2-NBDG delivery for 10 mM and 6 mM. Each group in panel B contains the same n = 7 subjects. p values are from a student's paired t-test. Error bars show standard error. Values in table are mean ± standard error. (C) R_D_ was strongly correlated with 2-NBDG fluorescence at 5 minutes (p<0.001). R_D_ did not correlate with 2-NBDG_60_ (not shown). (D) 2-NBDG_60_/R_D_ was inversely correlated with baseline blood glucose in normal mice (R = −0.61, p = 0.02). 2-NBDG_60_ was also correlated with blood glucose (R = −0.52, p = 0.05, not shown). For animals that received both 6 mM and 10 mM doses, the average values of the endpoints (2-NBDG_05_, 2-NBDG_60_, and 2-NBDG_60_/R_D_) for both doses were used in calculating the correlations. These subjects are denoted by “mean” in the legend. n = 15 mice for (C) and (D).

Fig. 2 describes the relationship between the rate of 2-NBDG kinetics and the administered 2-NBDG dose. Fig. 2A shows representative images of 2-NBDG uptake over 60 minutes in a normal mouse injected with either 6 mM 2-NBDG or 10 mM 2-NBDG on consecutive days. Fig. 2B summarizes the results of imaging 6 mM and 10 mM doses in the same cohort of mice. The table shows the ratio of endpoints comparing the 10 mM and 6 mM groups. Each ratio was calculated on a per-mouse basis, the ratios for each mouse were averaged, and values are presented as mean ratio ± standard error. The expected ratio of 10 mM/6 mM endpoints is 1.67 if all differences between groups are attributable to differences in injected dose. At 5 minutes post-injection, the fluorescence ratio of the dose groups (10 mM/6 mM) closely approached the expected ratio of 1.67 (p<0.01), indicating that early time points report primarily on delivery. The ratio of R_D_ (calculated as R_D(10 mM)_/R_D(6 mM)_) showed similar results.

We hypothesized that correcting 2-NBDG uptake for variations in R_D_ due to inter-mouse variation and injected 2-NBDG dose would better represent glucose uptake. First, in Fig. 2C we confirmed that while R_D_ and 2-NBDG uptake at 5 minutes post-injection (2-NBDG_05_) are highly correlated (R = 0.77, p<0.001), R_D_ and 2-NBDG at 60 minutes post injection are independent endpoints (R = 0.20, p = N.S., not shown). To validate that delivery-corrected 2-NBDG uptake more accurately represents glycolytic uptake, we investigated the correlation of 2-NBDG_60_/R_D_ with blood glucose concentration in normal mice. Fig. 2D shows a significant inverse correlation between 2-NBDG_60_/R_D_ and blood glucose (R = −0.61, p = 0.02).

### The rate of 2-NBDG delivery, R_D_, is positively correlated with blood velocity

**Figure 3:**
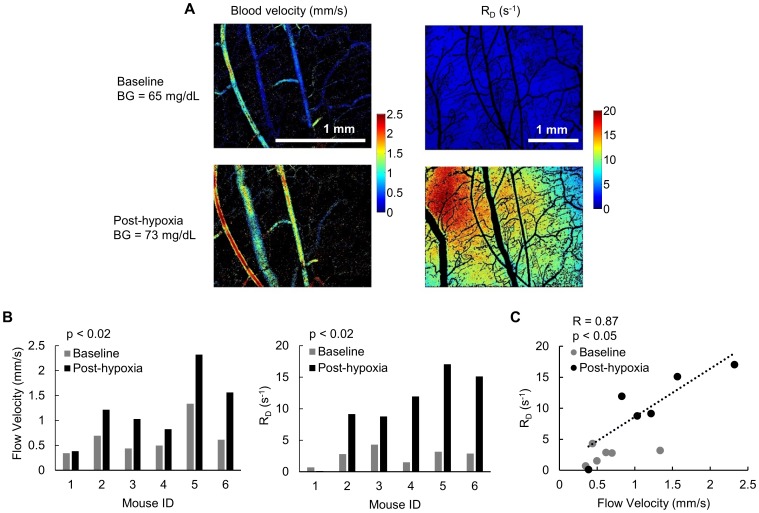
The rate of 2-NBDG delivery, R_D_, is strongly correlated with blood velocity. (A) Representative images of blood velocity and the rate of 2-NBDG delivery (R_D_) in a normal mouse at baseline and during reoxygenation after 1 hour of hypoxia. (B) Paired data for a set of mice at baseline and after 1 hour of hypoxia. After hypoxia, flow velocity and R_D_ increased significantly (p<0.02 for both). N = 6 mice. (C) The rate of 2-NBDG delivery (R_D_) is highly correlated with blood velocity (R = 0.87, p<0.05). The trendline corresponds to the trend for post-hypoxia data only.

The results presented in Fig. 3 show the relationship between red blood cell velocity and the rate of 2-NBDG delivery, R_D_, in corresponding image regions. Each mouse was imaged at baseline under normoxic condition (21% inspired O_2_) and after 10 minutes of re-oxygenation from breathing hypoxia (10% inspired O_2_). Mice were randomly assigned to undergo baseline or post-hypoxia (hyperemia) imaging first. Fig. 3A shows representative images of a mouse at baseline and after hypoxia. There is a clear increase in flow velocity as well as R_D_ after hypoxia. Fig. 3B shows that hypoxia was successfully used to significantly increase blood velocity in the tissue (p<0.02). Flow velocity increased in all mice after hypoxia. A corresponding significant increase in R_D_ was seen after hypoxia (p<0.02). Only one mouse did not show an increase in R_D_. In Fig. 3C, flow velocity and R_D_ show a strong correlation after hypoxia (R = 0.87, p<0.05). At baseline, the trend was similar, but the range of flow velocities was truncated compared to the group that underwent hypoxia.

### The ratio 2-NBDG_60_/R_D_ reflects stereo-specific uptake *in vivo*

**Figure 4:**
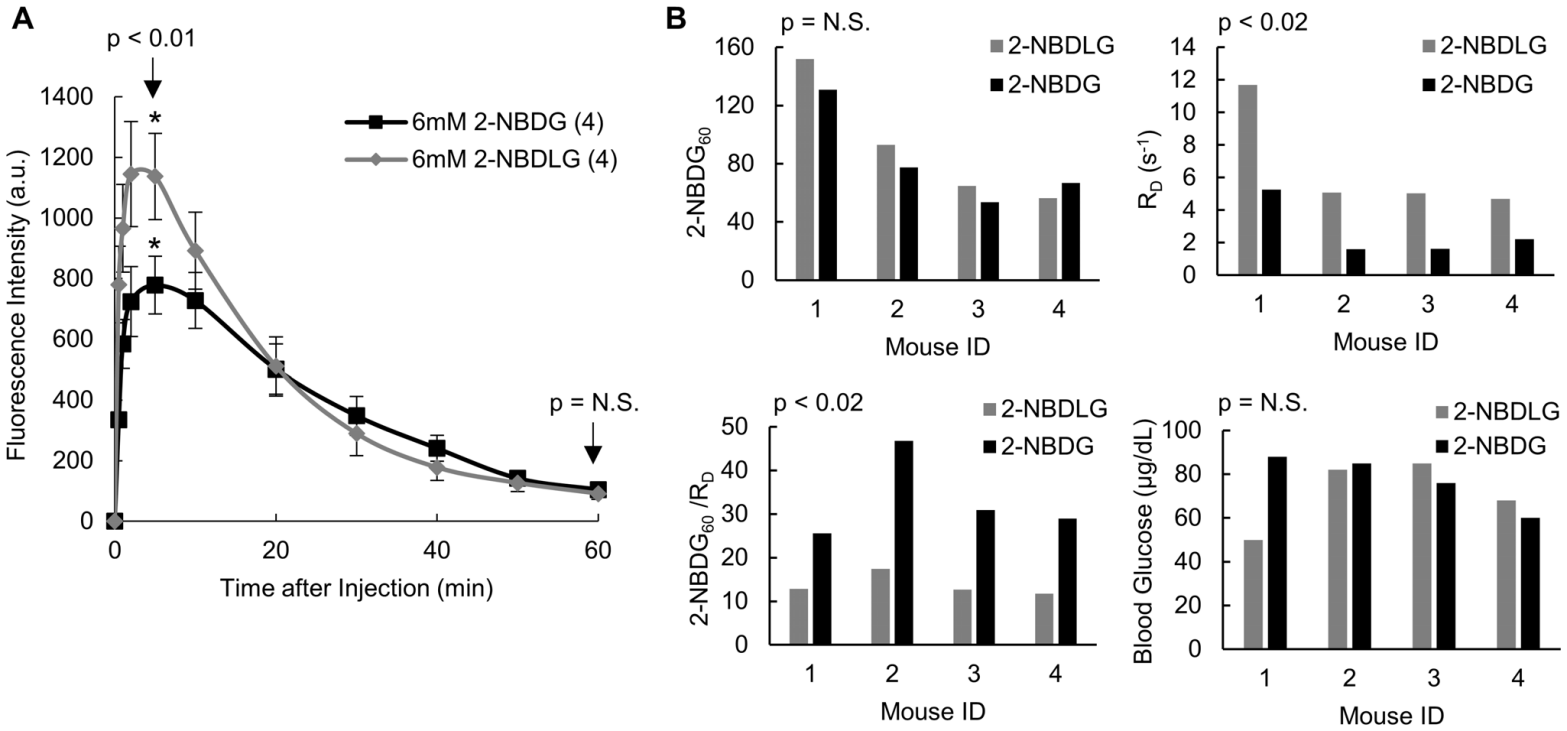
The ratio 2-NBDG_60_/R_D_ reflects stereo-specific uptake *in vivo*. (A) Mean kinetics of non-specific control (2-NBDLG) and specific tracer (2-NBDG) uptake imaged in the same cohort of mice on subsequent days. Peak fluorescence is significantly greater after 2-NBDLG administration than after 2-NBDG administration (p<0.01). Fluorescence at 60 minutes is comparable for both tracers (p = N.S.) (B) Results of paired tracer and control imaging in a set of four mice. Neither blood glucose nor 2-NBDG_60_ was significantly different between the two imaging perturbations (p = N.S.). R_D_ was greater for the control 2-NBDLG than for 2-NBDG (p<0.02). 2-NBDG_60_/R_D_ identifies specific tracer uptake, as it is significantly greater for 2-NBDG than 2-NBDLG (p<0.02). n = 4 mice. Each p-value represents results of a student's paired t-test.

Fig. 4 shows the kinetic profiles of 2-NBDG and the non-specific control 2-NBDLG in normal window chambers. Fig. 4A compares the uptake kinetics of the two tracers in a group of four non-tumor mice. Fluorescence intensity increased for both 2-NBDLG and 2-NBDG and peaked at 3–5 minutes. The time-to-peak (T_max_) did not vary significantly based on the administered tracer (p = N.S. (0.07), not shown). However, peak fluorescence (2-NBDG_max_) was significantly greater when 2-NBDLG was administered compared to 2-NBDG (p<0.01). It follows that the rate of delivery, R_D_ = 2-NBDG_max_/T_max_, was greater for 2-NBDLG than 2-NBDG (p<0.02). We have previously established that fluorescence at 60 minutes after injection corresponds predominantly to intracellular fluorescence [27]. Here, by 60 minutes post-injection, mean fluorescence intensities from the two compounds (2-NBDG_60_ and 2-NBDLG_60_) were indistinguishable between groups (p = N.S.). 2-NBDG uptake should exceed 2-NBDLG uptake, since 2-NBDG fluorescence represents stereo-specific uptake into the cell in addition to non-specific accumulation. The graphs in Fig. 4B shows a paired comparison of 2-NBDG and 2-NBDLG uptake properties in each of four mice. For each mouse, fluorescence at 60 minutes was similar for the two tracers (p = N.S. (0.27)). R_D_ was increased for 2-NBDLG relative to 2-NBDG in all mice (p<0.02). After correction for the increased delivery of 2-NBDLG, differences in specific and non-specific become apparent. In each mouse, 2-NBDG_60_/R_D_ was significantly greater than 2-NBDLG_60_/R_D_ (p<0.02), representing the difference in demand for the two tracers. Blood glucose did not vary significantly between imaging days (p = N.S.).

### Delivery-corrected glucose uptake reveals distinct glycolytic phenotypes in metastatic (4T1) and non-metastatic (4T07) mammary tumors

**Figure 5:**
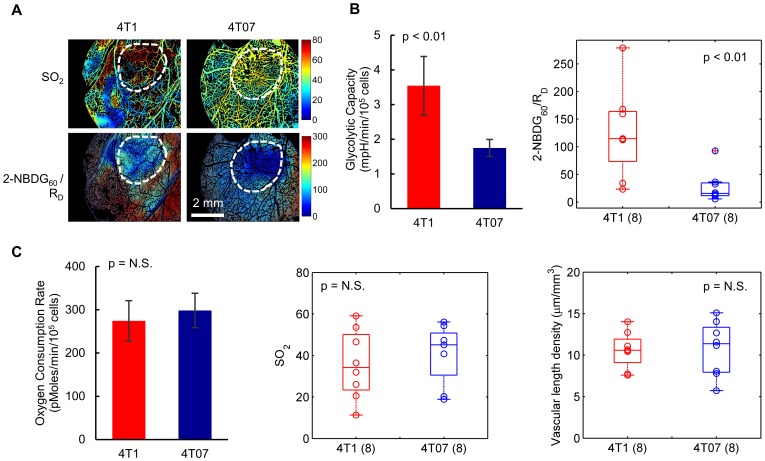
Delivery-corrected glucose uptake reveals distinct glycolytic phenotypes in metastatic (4T1) and non-metastatic (4T07) mammary tumors. (A) Representative images of vascular oxygen saturation (SO_2_) and delivery-corrected 2-NBDG (2-NBDG_60_/R_D_) for a 4T1 tumor and a 4T07 tumor, *in vivo*. (B) 2-NBDG_60_/R_D_ showed contrast in glucose uptake between metastatic 4T1 and non-metastatic 4T07 tumors *in vivo* (p<0.01). A Seahorse Glycolysis Stress Test also revealed that the glycolytic capacity, defined as extracellular acidification rate (ECAR) after blockade of respiration by oligomycin, was significantly greater for 4T1 than for 4T07 (p<0.01). (C) Mean vascular oxygen saturation (SO_2_) was comparable for 4T07 and 4T1 tumors in window chambers (p = N.S.). Vascular density was indistinguishable between tumor lines (p = N.S.). A Seahorse Glycolysis Stress Test showed that oxygen consumption rate (OCR) is comparable for 4T1 and 4T07 tumors (p = N.S.). Number of mice per group indicated by group name on axis. For Seahorse results, n = 12 cell samples from 3 distinct assays. Midline of box plots show median, box edges correspond to 25^th^ and 75^th^ percentiles, and scatter points show all data values.

**Figure 6:**
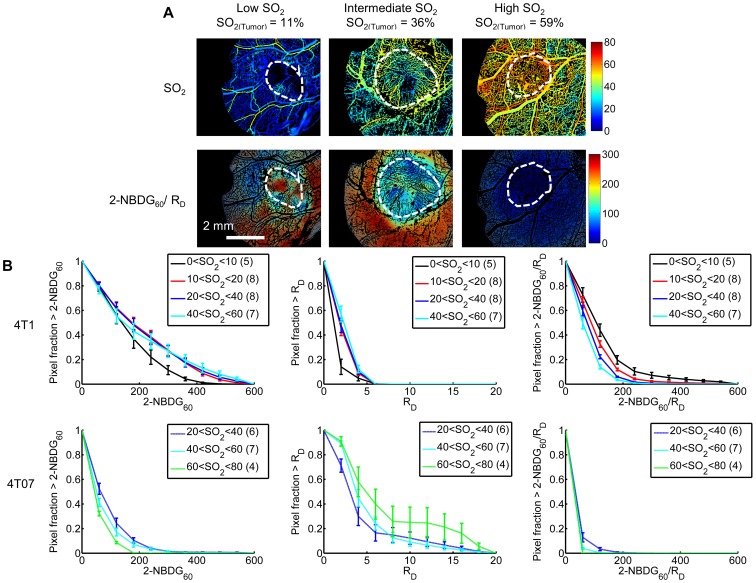
The ratio 2-NBDG/R_D_ facilitates assessment of glucose demand in heterogeneous regions of metastatic mammary tumors. (A) Representative images of vascular oxygenation (SO_2_) and delivery-corrected 2-NBDG (2-NBDG_60_/R_D_) for a 4T1 tumor with low mean SO_2_, a 4T1 tumor with intermediate mean SO_2_, and a 4T07 with high mean SO_2_. Adapted from Rajaram, et al. 2013. (B) Survival curves (1-cumulative distributions) show 2-NBDG_60_, R_D_, and 2-NBDG_60_/R_D_ for regions of distinct SO_2_ (%) in 4T07 and 4T1 tumors. For 4T1, 2-NBDG_60_ is lower for 0<SO_2,4T1_<10 regions than for any other SO_2,4T1_ (p = N.S.). Significantly lower rates of R_D_ are seen for the 0<SO_2,4T1_<10 group than for well-oxygenated 4T1 regions (p<0.05 or p<0.01 for 0<SO_2,4T1_<10 vs. 20<SO_2,4T1_<40 or 40<SO_2,4T1_<60, respectively). After correction for low R_D_, 2-NBDG_60_/R_D_ increased slightly but significantly in hypoxic regions (p<0.01 for 0<SO_2,4T1_<10 vs. 40<SO_2,4T1_<60). For 4T07, 2-NBDG uptake for the highest SO_2,4T07_ regions decreased compared to the lowest SO_2,4T07_ (p<0.01 for all 20<SO_2,4T07_<40 vs. 60<SO_2,4T1_<80). R_D_ is indistinguishable between SO_2,4T07_ levels. After correction by R_D_, 2-NBDG_60_/R_D_ is lowest for 60<SO_2,4T07_<80 (p<0.01). Comparison between 4T1 and 4T07 shows that 2-NBDG_60_ is higher for all SO_2,4T1_ than all SO_2,4T07_ (p<0.01). On the other hand, R_D_ for the best oxygenated 4T07 groups (40<SO_2,4T07_<60 and 60<SO_2,4T07_<80) is greater than for all 4T1 groups (p<0.01 for all groups except 40<SO_2,4T1_<60 vs. 60<SO_2,4T07_<80 where p<0.06). After correction by R_D_, 2-NBDG_60_/R_D_ is higher for all SO_2,4T1_ than all SO_2,4T07_ (p<0.01 for all SO_2,4T1_ compared to all SO_2,4T07_). Number of mice per group indicated by group name in legend.

We used 2-NBDG_60_/R_D_ to compare tumors with different metabolic phenotypes: metastatic 4T1 tumors and nonmetastatic 4T07 tumors. Fig. 5 shows representative images of SO_2_ and 2-NBDG_60_/R_D_ from window chambers with 4T1 or 4T07 tumors. Fig. 5B shows that averaging over the entire tumor regions (or regions of normal tissue) resulted in a significantly higher 2-NBDG_60_/R_D_ for 4T1 than for 4T07 (p<0.01).. A Seahorse Glycolysis Stress Test was used on 4T1 and 4T07 cells to compare with the results of *in vivo* metabolic imaging. The glycolytic capacity, defined as the extracellular acidification rate (ECAR) after blockade of respiration by oligomycin, was significantly greater for 4T1 than for 4T07. These results are consistent with the intravital microscopy data, in which 4T1 tumors took up significantly more 2-NBDG than 4T07 tumors, both on average (Fig. 5B) and at each SO_2_ level (Fig. 6B).

An elevated level of glucose uptake may lead to the assumption that the tissue is hypoxic and therefore increasingly dependent on glycolysis, but no significant difference in SO_2_ was seen between groups (Fig. 5C). Additionally, vascular density, the total length of vessels per unit volume, was indistinguishable between 4T1 and 4T07 tumors, implying that differences in SO_2_ may be attributable to changes in oxygen consumption. Results of the Seahorse Glycolytic Stress Test show that oxygen consumption rate (OCR) is comparable for 4T1 and 4T07 tumors (p = N.S.).

### The ratio 2-NBDG_60_/R_D_ facilitates assessment of glucose uptake in heterogeneous regions of metastatic mammary tumors

Tumor oxygenation plays an important role in metabolism, and varies not only across tumor lines but also within a tumor [36], [37]. We parsed our delivery-corrected glucose demand endpoint with vascular oxygenation to investigate metabolic heterogeneity in tumors. First we compared 4T1 and 4T07 tumors with mean vascular oxygenation values in different SO_2_ ranges, shown in Fig. 6A. After correcting for delivery, the hypoxic 4T1 tumor (mean SO_2_ = 11%) showed localized regions of high 2-NBDG_60_/R_D_ uptake not seen in the 4T1 with intermediate SO_2_ (mean SO_2_ = 36%) nor in the well-oxygenated 4T07. The well-oxygenated 4T07 tumor (mean SO_2_ = 59%) showed an appreciably decreased 2-NBDG_60_/R_D_ compared to either of the 4T1 tumors. The emerging trend suggested that 2-NBDG/R_D_ increased as average SO_2_ decreased.

We then analyzed each tumor at five levels of vascular oxygenation (SO_2_) to identify if hypoxic regions were responsible for increased mean glucose uptake in 4T1 tumors relative to 4T07 tumors. Fig. 6B shows 2-NBDG_60_, R_D_, and 2-NBDG_60_/R_D_ for 4T07 tumors and 4T1 tumors, respectively, across vascular oxygenation levels: 0–10% SO_2_, 10–20% SO_2_, 20–40% SO_2_, 40–60% SO_2_, and 60–80% SO_2_. Each curve represents the mean of distributions at a given SO_2_ level from up to 8 mice (group numbers listed in parentheses in legend). Interestingly, only two 4T07 mice exhibited vessels with the lowest levels of oxygenation (0–10% SO_2_ and 10–20% SO_2_), and therefore were not shown. Similarly, only two 4T1 mice exhibited vessel regions of 60–80% SO_2_ and were therefore excluded.

Within the 4T1 tumors, hypoxic regions had decreased 2-NBDG delivery compared to well-oxygenated regions (p<0.01, 0–10% SO_2_ v. 40–60% SO_2_). There was no difference in uptake between other SO_2,4T1_ groups. R_D_ was also lowest in hypoxic regions of 4T1 (p<0.05 or p<0.01 for 0<SO_2,4T1_<10 vs. 20<SO_2,4T1_<40 or 40<SO_2,4T1_<60, respectively). The ratio 2-NBDG_60_/R_D_ within 4T1 significantly decreased as vascular oxygenation increased reflecting the Pasteur effect (p<0.01 for 0<SO_2,4T1_<10 vs. 40<SO_2,4T1_<60). 4T07 tumors showed a different trend in uptake. 2-NBDG_60_ increased from the highest to the lowest SO_2_ levels of 4T07 tumors (p<0.01 for 20<SO_2,4T07_<40 vs. 60<SO_2,4T07_<80) and there was no difference in R_D_ across SO_2,4T07_ levels. After correction, 2-NBDG_60_/R_D_ in 4T07 followed a similar trend as in 4T1. 2-NBDG_60_/R_D_ was lowest for 60<SO_2,4T07_<80 compared to both other SO_2,4T07_ (p<0.01).

Comparison between tumor lines showed that 2-NBDG_60_ was higher for all 4T1 groups than for all 4T07 (p<0.01). On the other hand, delivery (R_D_) for the best oxygenated 4T07 groups (40<SO_2,4T07_<60 and 60<SO_2,4T07_<80) was greater than for all 4T1 groups (p<0.01 for all groups except 40<SO_2,4T1_<60 vs. 60<SO_2,4T07_<80 where p<0.06). At all SO_2_ levels, 2-NBDG_60_/R_D_ of 4T1 tumors exceeded that of 4T07 tumors (p<0.01 for all SO_2,4T1_ compared to all SO_2,4T07_). This analysis confirmed that 4T1 tumors display increased glucose metabolism regardless of oxygen status, not only in response to hypoxia. On the other hand, the low demand for and sufficient delivery of 2-NBDG to 4T07 made them statistically indistinguishable from normal tissue (not shown).

## Discussion

Previously, our group determined that the *in vivo* rate of 2-NBDG delivery has significant effects on the uptake of 2-NBDG, and as a result, the perceived glucose uptake of the tissue [27]. We have here presented a method of utilizing the kinetic profile of 2-NBDG uptake to correct for variations in delivery and uncover more accurately the glycolytic uptake *in vivo*. To validate our method of delivery correction, we first showed that the rate of delivery was significantly correlated with delivery-linked variables. Varying the injected concentration of 2-NBDG from 6 mM to 10 mM was sufficient to cause an increase in the rate of delivery, due to an increased 2-NBDG fluorescence at 5-minutes after injection. Upon further investigation of the 6 mM and 10 mM cohorts, we found that there was no difference in T_max_ between groups (p = 0.50, not shown). The difference in R_D_ was explained by a difference in the intensity 2-NBDG_max_ between groups, which was expected to vary with injected dose.

We further wished to show that R_D_ varies with variations in the time of delivery. Hypoxia has been shown to increase red blood cell velocity in normal tissue, for example rat brain [38], which we hypothesized would cause a resulting increase in the delivery speed of 2-NBDG. The injected concentration of 2-NBDG was kept constant at 6 mM to avoid confounding effects. We found that one hour of breathing hypoxic gas (10% O_2_, balance N_2_) followed by 10 minutes of breathing room air was sufficient to increase the velocity of red blood cells in normal (non-tumor) vasculature. As hypothesized, we saw a corresponding increase in the rate of 2-NBDG delivery. Prior to hypoxia, the range of blood flow velocities was not sufficient to obtain a wide range of 2-NBDG delivery rates. Hyperemia significantly increased the blood velocity range allowing for an improved correlation with R_D_ (R = 0.87, p<0.05). This is not surprising because metabolic substrate delivery is tightly correlated to demand in normal tissue [39], [40] and glucose demand is increased by hypoxia [41].

To investigate whether correction for delivery effects altered the relationship between glucose and 2-NBDG, we calculated the correlations between 2-NBDG_60_ and blood glucose concentration and between 2-NBDG_60_/R_D_ and blood glucose concentration. A moderate inverse correlation was seen between 2-NBDG_60_/R_D_ and blood glucose concentration (R = −0.61, p = 0.02) as well as between 2-NBDG_60_ and blood glucose concentration (R = −0.52, p = 0.05, not shown), indicating that 2-NBDG competition with blood glucose is real, and not an artifact of delivery correction. The trends we observed are consistent with *in vitro* studies of oral neoplasia showing that 2-NBDG uptake is competitively inhibited by glucose, and 2-NBDG fluorescence decreases with increasing glucose concentration [19]. Further, 2-NBDG uptake has been shown to increase with an increase in glucose demand. For example, Sheth and colleagues showed *in vivo* that 2-NBDG uptake in the brain increases greatly during a seizure, a well-established instance of increased glucose demand [23]. In accordance with these findings, we now show that blood glucose competes with 2-NBDG *in vivo*, and caution that major variations in blood glucose may change the interpretation of 2-NBDG data.

We then asked if 2-NBDG_60_/R_D_ was capable of distinguishing between controlled instances of varied tracer uptake *in vivo*. We used 2-NBDG in unperturbed normal tissue *in vivo*, taking advantage of the baseline level of glucose demand. To simulate a contrasting situation of negligible demand *in vivo*, we used the fluorescent molecule 2-NBDLG, which has been developed for use as a 2-NBDG control substance [29]. Though identical in molecular weight and fluorescent spectrum to 2-NBDG, 2-NBDLG is unrecognized by the GLUT receptors and cannot be actively transported into the cell. Instead, 2-NBDLG fluorescence may represent non-specific adsorption onto the cell membrane or uptake through damaged membrane [29]. 2-NBDLG fluorescence may also correspond to tracer accumulation in the interstitial space, though we have shown that the specific probe 2-NBDG clears the interstitial space by 60 minutes after injection [27]. We hypothesized that, for a given animal, uptake of 2-NBDG would exceed uptake of 2-NBDLG. Interestingly, 2-NBDG and 2-NBDLG fluorescence intensities at 60 minutes were indistinguishable in each animal. In a separate experiment, we found that the average fluorescent intensity of 100 nM 2-NBDLG in solution was approximately 25% greater than the fluorescent intensity of 100 nM 2-NBDG (data not shown), indicating a greater fluorescence quantum yield for the control solution. We caution that care must be taken to properly calibrate for differences in fluorescent behavior when using a control marker for 2-NBDG *in vitro* or *in vivo*. Our results showed that our correction by R_D_ was able to account for the difference in fluorescence intensity between 2-NBDLG and 2-NBDG. After correcting for a greater max intensity of 2-NBDLG, 2-NBDG accumulation was approximately 3-fold higher than 2-NBDLG accumulation in all animals, indicating that we were able to identify stereo-specific uptake.

Since metabolic substrate delivery is tightly controlled in normal tissue [39], [40], external perturbations were needed to observe significant changes in delivery and demand. This allowed us to validate our method in a controlled way. However, our ultimate goal was to utilize our strategy to characterize tumors, where delivery and demand may be “mis-matched”. Using PET to find discrepancies between blood flow and FDG delivery has proven useful for characterizing disease in heart and brain tissue [42], [43]. Specht, et al. were among the first to use functional imaging to uncover an altered relationship between tumor metabolism and blood flow that existed in breast cancer subtypes [28]. We, too, hypothesized that our method correlated to the long-term fate of different tumor subtypes- in particular, metastatic potential. As previously mentioned, 4T1 is a metastatic murine mammary tumor line, and 4T07 is a non-metastatic sister murine mammary tumor line [30], [31]. An assessment of 2-NBDG_60_/R_D_ values averaged over the entire tumor regions revealed that glycolytic uptake of the 4T1 tumors far exceeded that of 4T07 tumors, as shown by our previous results [27] and now corroborated by a Seahorse assay.

We additionally showed that these differences are not due to differences in oxygenation, as average SO_2_, vascular density and oxygen consumption rate were comparable between 4T1 and 4T07 tumors. Our *in vivo* and Seahorse *in vitro* results are consistent with previous work showing that lactate concentration in tumors both fuels tumor growth and is predictive of metastasis [44]–[47]. Recently, Sonveaux et. al have proposed a mechanism which may underlie the association between lactate and tumor aggressiveness. They showed that lactate upregulates HIF-1 in endothelial cells, and that blocking lactate entry through monocarboxylate transporter 1 can prevent endothelial migration and tumor angiogenesis [48].

Lastly, we wanted to investigate regional trends in 2-NBDG uptake, as it is well established that the tumor microenvironment is highly heterogeneous with respect to oxygenation [36], [37], [49]. For example, vascular remodeling in tumors leads to areas of decreased oxygen delivery to the cells. Studies in window chambers have shown that tumor tissue can approach anoxia as close as 100 um from a vessel [50]. However, cells are often able to compensate by increasing glucose uptake for use in glycolysis [41]. We would then expect 2-NBDG uptake to increase as SO_2_ decreases. A combination of low 2-NBDG uptake and low SO_2_, however, may indicate non-viable cells chronically starved of both glucose and oxygen [51], [52]. Modeling has shown that glucose diffuses farther than oxygen [53] and that glucose concentration decreases only slightly over a distance of ∼30 cells from a vessel [54]. We excluded pixels farther than 150 µm from a vessel to ensure that non-viable cells were not mischaracterized.

Interestingly, we observed in 4T1 that 2-NBDG_60_ uptake was lowest in regions of very low oxygenation. Looking at R_D_ reveals that diminished delivery contributes to low levels of 2-NBDG uptake in hypoxic regions. After correcting for decreased delivery to viable cells, the poorly oxygenated 4T1 regions showed elevated 2-NBDG_60_/R_D_ compared to tumor's well-oxygenated regions. On the other hand, 4T07 tumors did not exhibit hypoxic regions nor regions of poor 2-NBDG delivery. As in 4T1 tumors, 2-NBDG_60_/R_D_ increased significantly as vascular oxygenation decreased in 4T07.

In addition to sustaining the highest 2-NBDG_60_ at all oxygenation levels, the 4T1 tumors also had lower R_D_ than 4T07 tumors or normal tissue. For our dataset, a lower R_D_ corresponded to a longer 2-NBDG delivery time-to-max, indicating impeded delivery (mean T_max,4T1_ = 10.88 min, mean T_max,4T07_ = 7.20 min, mean T_max,norm_ = 4.42 min). Tumors often have impeded delivery of nutrients due to the immature and tortuous vessels created by angiogenesis [37]. Interestingly, some tumors with long capillary transport times adapt by upregulating aerobic glycolysis [55]. This type of Warburgian metabolism would be consistent with our findings for 4T1, which had sustained high glucose uptake across oxygen levels.

As a last consideration, it is important to note that our strategy may be particularly effective in regions of poor delivery, identified by slow blood velocity or hypoxia. Mankoff and colleagues have demonstrated with FDG-PET that a mismatch between tumor metabolism and blood flow, in particular high metabolic rate relative to blood flow, is an indicator of poor prognosis in tumors [56]. For this reason, we have developed our method to help us quickly identify tumor regions with poor delivery but sustained 2-NBDG uptake. Additionally, hypoxia is an indicator of poor prognosis in regard to treatment response, recurrence, and overall outcome [57]–[59], so the ability to identify hypoxic tumor regions is crucial. With further development, our method of imaging delivery-corrected 2-NBDG uptake and oxygenation is well-poised as a tool for pre-clinical and clinical tumor characterization.
